# The Distribution of Henipaviruses in Southeast Asia and Australasia: Is Wallace’s Line a Barrier to Nipah Virus?

**DOI:** 10.1371/journal.pone.0061316

**Published:** 2013-04-24

**Authors:** Andrew C. Breed, Joanne Meers, Indrawati Sendow, Katharine N. Bossart, Jennifer A. Barr, Ina Smith, Supaporn Wacharapluesadee, Linfa Wang, Hume E. Field

**Affiliations:** 1 Epidemiology, Surveillance and Risk Group, Animal Health and Veterinary Laboratories Agency, Addlestone, Surrey, United Kingdom; 2 School of Veterinary Science, University of Queensland, Brisbane, Queensland, Australia; 3 Balai Besar Penelitian Veteriner, Bogor, West Java, Indonesia; 4 Australian Animal Health Laboratory, CSIRO Livestock Industries, East Geelong, Victoria, Australia; 5 Neuroscience Center for Research and Development, Faculty of Medicine Chulalongkorn University, Bangkok, Thailand; 6 Biosecurity Queensland, Department of Employment, Economic Development and Innovation, Brisbane, Queensland, Australia; Thomas Jefferson University, United States of America

## Abstract

Nipah virus (NiV) (Genus Henipavirus) is a recently emerged zoonotic virus that causes severe disease in humans and has been found in bats of the genus *Pteropus*. Whilst NiV has not been detected in Australia, evidence for NiV-infection has been found in pteropid bats in some of Australia’s closest neighbours. The aim of this study was to determine the occurrence of henipaviruses in fruit bat (Family Pteropodidae) populations to the north of Australia. In particular we tested the hypothesis that Nipah virus is restricted to west of Wallace’s Line. Fruit bats from Australia, Papua New Guinea, East Timor and Indonesia were tested for the presence of antibodies to Hendra virus (HeV) and Nipah virus, and tested for the presence of HeV, NiV or henipavirus RNA by PCR. Evidence was found for the presence of Nipah virus in both *Pteropus vampyrus* and *Rousettus amplexicaudatus* populations from East Timor. Serology and PCR also suggested the presence of a henipavirus that was neither HeV nor NiV in *Pteropus alecto* and *Acerodon celebensis*. The results demonstrate the presence of NiV in the fruit bat populations on the eastern side of Wallace’s Line and within 500 km of Australia. They indicate the presence of non-NiV, non-HeV henipaviruses in fruit bat populations of Sulawesi and Sumba and possibly in Papua New Guinea. It appears that NiV is present where *P. vampyrus* occurs, such as in the fruit bat populations of Timor, but where this bat species is absent other henipaviruses may be present, as on Sulawesi and Sumba. Evidence was obtained for the presence henipaviruses in the non-Pteropid species *R. amplexicaudatus* and in *A. celebensis*. The findings of this work fill some gaps in knowledge in geographical and species distribution of henipaviruses in Australasia which will contribute to planning of risk management and surveillance activities.

## Introduction

Hendra virus (HeV) and Nipah virus (NiV) are paramyxoviruses of the genus *Henipavirus,* and bats of the Genus *Pteropus* (Family Pteropodidae) have been identified as their primary wildlife reservoir [1]. These viruses have repeatedly spilled over from the reservoir hosts to cause disease in domestic animals and humans in Australia, Malaysia, Bangladesh and India [2]. Considerable effort has been expended to determine the distribution of henipaviruses and the bat species that constitute reservoir hosts for HeV and NiV. Serological evidence of infection has been found in 28 species, 12 from the Genus *Pteropus* (see Table 1). Despite this effort, there are few published accounts of isolation of henipaviruses from wild bats. These include: three isolates of HeV from *Pteropus poliocephalus* [3]; four isolates of HeV from *Pteropus alecto* [4]; one isolate of HeV from *Pteropus conspicillatus* [4]; and single isolates of NiV from *Pteropus hypomelanus*, *Pteropus lylei* and *Pteropus vampyrus* [5]–[7].

**Table 1:** Published detections of henipaviruses in bat species.

Location	Bat species	Virus	Test	Reference
Australia	*Pteropus alecto*	Hendra	Isolation	[44]
Australia	*Pteropus conspicillatus*	Hendra	Isolation	[4]
Australia	*Pteropus poliocephalus*	Hendra	Isolation	[44]
Australia	*Pteropus scapulatus*	Hendra	Serology VNT	[45]
Bangladesh	*Pteropus giganteus*	Nipah	Serology ELISA	[46]
Cambodia	*Pteropus lylei*	Nipah	Isolation	[47]
China	*Hipposideros pomona*	Nipah-like	Serology ELISA	[41]
China	*Miniopterus spp.*	Nipah-like	Serology western blot	[41]
China	*Myotis daubentonii*	Nipah-like	Serology western blot	[41]
China	*Myotis ricketti*	Nipah-like	Serology western blot	[41]
China	*Rhinolophus affinis*	Nipah-like	Serology western blot	[41]
China	*Rhinolophus sinicus*	Nipah-like	Serology ELISA	[41]
China	*Rousettus leschenaulti*	Nipah-like	Serology western blot	[41]
Equatorial Guinea	*Eidolon helvum*	Henipavirus	Serology VNT	[48]
Ghana	*Eidolon helvum*	Henipavirus	PCR	[10]
Ghana	*Epomophorus gambianus*	Henipavirus	Serology Luminex	[49]
Ghana	*Hypsignathus monstrosus*	Henipavirus	Serology Luminex	[49]
India	*Pteropus giganteus*	Henipavirus	Serology VNT	[50]
Indonesia – Kalimantan	*Pteropus vampyrus*	Nipah	Serology VNT	[18]
Indonesia – Sumatra Java	*Pteropus vampyrus*	Henipavirus	Serology VNT	[18]
Madagascar	*Eidolon dupreanum*	Nipah	Serology VNT	[51]
Madagascar	*Pteropus rufus*	Nipah	Serology VNT	[51]
Malaysia	*Cynopterus brachyotis*	Nipah	Serology VNT	[52]
Malaysia	*Eonycteris spelaea*	Nipah	Serology VNT	[52]
Malaysia	*Pteropus hypomelanus*	Nipah	Isolation	[53]
Malaysia	*Pteropus vampyrus*	Nipah	Isolation	[54]
Malaysia	*Scotophilus kuhlii*	Nipah	Serology VNT	[52]
Papua New Guinea	*Dobsonia andersoni*	Hendra	Serology	[20]
Papua New Guinea	*Dobsonia magna*	Hendra	Serology	[20]
Papua New Guinea	*Pteropus admiralitatum*	Hendra	Serology	[20]
Papua New Guinea	*Pteropus capistratus*	Hendra	Serology	[20]
Papua New Guinea	*Pteropus hypomelanus*	Hendra	Serology	[20]
Papua New Guinea	*Pteropus neohibernicus*	Hendra	Serology	[20]
Thailand	*Hipposideros larvatus*	Nipah	Serology ELISA	[55]
Thailand	*Pteropus hypomelanus*	Nipah	Serology ELISA	[55]
Thailand	*Pteropus lylei*	Nipah	PCR	[55]
Thailand	*Pteropus vampyrus*	Nipah	Serology ELISA	[55]
Vietnam	*Rousettus leschenaulti*	Nipah	Serology western blot	[42]
Vietnam	*Cynopterus sphinx*	Nipah	Serology western blot	[42]

Evidence of henipavirus infection has been found across the range of *Pteropus* bats from eastern Australia, north to Indonesia, Malaysia, Thailand and Cambodia; and west to Bangladesh, India and Madagascar, suggesting that these viruses occur throughout the geographic range of this genus [8]. Henipavirus infection has also been found to be present in *Eidolon helvum*, a species of fruit bat occurring throughout sub-Saharan Africa [9], [10]. Given that over two billion people live in the area where *Pteropus* and *Eidolon* bats are present, even sporadic or occasional spillover of virus from bats to humans may result in a significant number of human infections.

Hendra virus has spread from *Pteropus* bats to horses in Australia on at least 33 separate occasions, always with fatal consequences [4]. Seven humans who have had close contact with infected horses have become infected with HeV, including four fatally [11]–[13]. While the economic and public health consequences of Hendra virus have been limited to date, the effects of Nipah virus have been much more severe. Nipah virus was responsible for an outbreak of disease in pigs and humans in peninsular Malaysia and Singapore in 1998–1999 resulting in the death of over 100 people and the culling of over one million pigs [14]. Since that time there have been at least 10 outbreaks of NiV disease in humans in Bangladesh and India with the resultant death of over 140 people [15]; there has also been clear evidence of human to human transmission of this virus indicating potential for a human epidemic [16].

The apparent distribution of HeV and NiV is currently separated by the biogeographic region known as Wallacea, with the presence of NiV confirmed by viral isolation and/or PCR from *P. hypomelanus*, *P. lylei and P. vampyrus* from peninsular Malaysia and Cambodia (Table 1), and apparent on the basis of serological evidence from *P. vampyrus* on Sumatra, Java and Borneo [17], [18]. The presence of Hendra virus has been confirmed by viral isolation from *P. alecto, P. poliocephalus* and *P. conspicillatus* from Australia [4], [19], and is apparent based on serology in *P. scapulatus* in Australia [1] and *P. hypomelanus*, *P. neohibernicus*, *P. capistratus*, *P. admiralitatum*, *Dobsonia magna* and *Dobsonia andersoni* from Papua New Guinea [20]. It is not known whether the distributions of HeV and NiV are mutually exclusive or overlap, or indeed if other henipaviruses exist between the locations where HeV and NiV occur. It is possible that HeV and NiV are relatively host species-specific, and that this has resulted in the apparent lack of overlap of the two viruses. Furthermore it may be that there is some form of competitive exclusion of one virus by the other from each of the two respective regions.

It has been known for well over 100 years that a major biogeographic barrier exists between the Australo-Papuan and Wallacean region on the one hand, and southeast Asia on the other, with different groups of both terrestrial vertebrates and invertebrates occurring on either side of this ‘line’ [21]. It has even been suggested that this boundary has protected Australia from the recent H5N1 avian influenza epidemic [22]. Of the major groups of terrestrial mammals, only rodents and bats extend across this region from southeast Asia into Australia. There are 13 species of Old World fruit bat (Family Pteropodidae) that occur only to the west of Wallace’s Line and 67 species that are confined to the east, while 20 species have wide distributions throughout the region and occur on both sides of the line [23].

The aim of this study is to investigate the occurrence of henipaviruses in fruit bat populations in the regions of northeast Australia (Queensland), New Guinea (Papua New Guinea) and Wallacea (Indonesia and East Timor). In particular we tested the hypothesis that Nipah virus is restricted in distribution to west of Wallace’s Line.

Fruit bats (Family Pteropodidae) were sampled from northeast Australia, Papua New Guinea (Western Province and Madang Province), East Timor (Cova Lima Province) and Indonesia (Sulawesi and Sumba), and tested for the presence of anti-Hendra virus (HeV) and anti-Nipah virus (NiV) antibodies. PCR tests were also conducted to determine the presence of henipavirus RNA.

## Materials and Methods

### Ethics Statement

All animal work followed the guidelines of the American Society of Mammalogists and the National Health and Medical Research Council of Australia [24], [25]. The study was approved by the Animal Ethics Committee of the Queensland Department of Primary Industries and Fisheries (Permit number FN 47/2003-1) and the Queensland Parks and Wildlife Service (Permit number WISP03721106).

The virus neutralisation test (VNT) results from 66 of the 109 fruit bats from Papua New Guinea presented here have been previously reported in Breed et al. [26]. The analytical approach presented in this study is novel and different to that reported in the previous study.

### Study Sites

Bats were sampled from the following locations: Townsville and Cairns, Queensland Australia; areas around Bensbach and Mabudawan, Western Province, Papua New Guinea; Madang township, Madang Province, Papua New Guinea; areas around Suai, Cova Lima District, East Timor; Waikabubak area, Sumba, Indonesia; areas around Manado, North Sulawesi Province, Indonesia.

### Capture and Sampling

Bats were caught in 12 m or 18 m mist nets suspended between two 12 m poles and anaesthetised for collection of samples. In Australia inhalation anaesthesia, delivering isoflurane (Isoflurane, Laser Animal Health Pty Limited) and oxygen via an anaesthetic machine, was used following the protocol described by Jonsson et al. [27]. In East Timor, Papua New Guinea, and Indonesia, bats were anesthetised using a combination of ketamine (Ketamil, Ilium, Smithfield, Australia) and medetomidine (Domitor, Novartis, Pendle Hill, Australia) injected into the pectoral muscles at similar doses to those used by Middleton et al. [28]. Atipamezole was used to reverse the effects of medetomidine. Blood samples were collected by venepuncture of the propatagial vein and aspiration of blood with a 23 or 25 gauge needle and 1 mL or 3 mL syringe depending on the size of the animal. Blood was allowed to clot in 2 mL tubes for 24 hours before centrifugation and separation of serum and storage at 4°C until testing. Samples of urine and saliva were collected onto cotton swabs and stored in viral transport media or an RNA stabilisation reagent (RNAlater, Qiagen, Doncaster, Australia) for the detection of viral RNA by RT-PCR. Each individual bat from Australia, Papua New Guinea, East Timor and Sumba has samples of urine, saliva and blood tested by PCR, while the urine samples from bats in Sulawesi were pooled with samples from five to 10 individuals per pool.

### Serological Tests

The currently accepted reference procedure for detection of antibodies to HeV and NiV is the VNT according to Daniels et al. and the OIE [29], [30]. This was performed on the serum samples at the Australian Animal Health Laboratory in Geelong, Victoria, Australia, which is the World Organisation for Animal Health (OIE) reference laboratory for HeV and NiV viruses. A serum sample was considered positive if it neutralised HeV or NiV at a dilution of 1∶5 or greater in the VNT. According to the OIE Manual of Diagnostic Tests and Vaccines for Terrestrial Animals “Anti-HeV antiserum neutralises HeV at an approximately four-fold greater dilution than that which neutralises NiV to the same extent. Conversely, anti-NiV antiserum neutralises NiV approximately four times more efficiently than HeV [14], [30].” Hence we categorised sera as reacting to HeV or NiV if a four-fold difference in titre was observed, or equivocal if comparative titres were equal or showed a two-fold difference. Luminex binding and inhibition serological assays were also performed on sera where sufficient sample volumes were available according to Bossart et al. [31].

### Viral RNA Detection (PCR) Tests

Presence of HeV and NiV nucleic acid was tested for by real-time reverse-transcriptase (RT) PCR assay (RT-qPCR) (TaqMan) according to Smith et al. [32] (HeV M gene) and Guilllaume et al. [33] (NiV N gene) at the Australian Animal Health Laboratory. Additionally, samples from Indonesia and Papua New Guinea were tested for henipavirus RNA using a consensus RT-qPCR assay for the N gene according to Feldman et al. [34] at Queensland Health Forensic and Scientific Services for Public Health Virology. As these assays were in a developmental stage at the time the work was conducted, strict cycle threshold (CT) cut-off values were not available. However, it was generally considered that samples with CT values: <35 were positive; 35–45 were ‘suspect’ positive, and >45 were negative. Samples returning ‘suspect’ positive results were not tested further.

Further to this, a subset of samples from East Timor and Papua New Guinea were tested for NiV nucleic acid using a duplex nested conventional RT-PCR for the N gene according to Wacharapluesadee et al. [35]. A sample was considered positive if a band of appropriate size was visualised. All samples producing such bands were sequenced to determine their genetic relationship to known henipavirus isolates.

Virus isolation was attempted from samples collected into viral transport media from which positive PCR results were obtained where qPCR indicated an adequate amount of viral material to be present.

## Results

Fruit bats were sampled for the presence of henipaviruses in Australia (Townsville and Cairns in Queensland), Papua New Guinea (Western Province and Madang Province), East Timor (Cova Lima Province) and Indonesia (north Sulawesi and Sumba), all of which are located to the east of Wallace’s Line. The following results were obtained from the various geographic locations:

### Australia

Sixty-four *P. alecto* were sampled near Townsville in January 2005 following detection of HeV in a horse in December 2004. HeV RNA was detected in the blood, urine and saliva of one sub-adult male *P. alecto* by RT-qPCR at CT values 35.3, 37.6 and 39.9 respectively. This animal tested negative for the presence of HeV antibodies by VNT. Antibodies to HeV were detected in 28 of the 64 (44%, 95% CI 32–56) animals tested. Comparative HeV-NiV titres were not performed on these sera.

**Figure 1:**
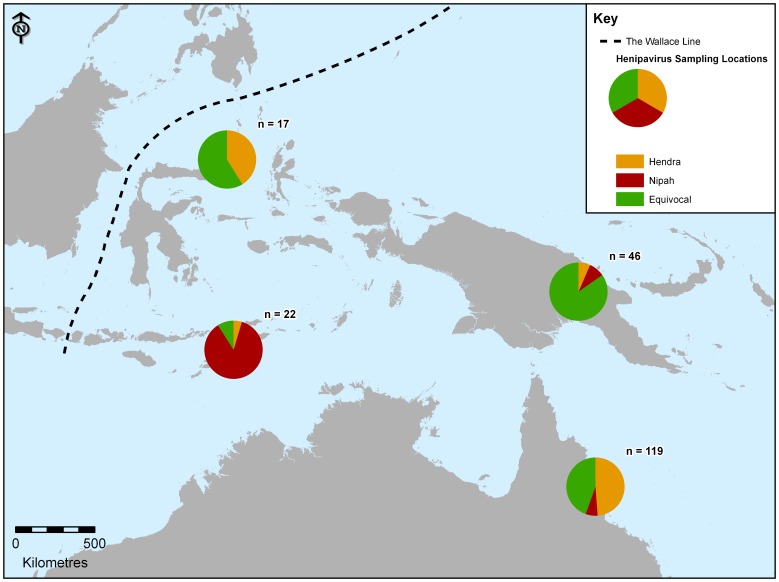
Comparative Hendra Virus and Nipah Virus Neutralisation Test results from Cairns, Papua New Guinea, Sulawesi and East Timor.

One-hundred-and-eighty *P. conspicillatus* were sampled near Cairns in June and November of 2005 following spillover of HeV to a horse and subsequently a human in October 2004. Neither HeV nor NiV RNA was detected in any of the animals sampled. Antibodies to HeV, NiV or both viruses were detected by VNT in 119 of 180 (66%, 95% CI 59–73) animals sampled. Of the animals testing positive on VNT, based on neutralising antibody titre, 52 (43.7%) indicated exposure to HeV, 8 (6.7%) indicated exposure to NiV, and 59 (49.5%) showed equivocal titres (see Table 2; Figure 1).

**Table 2:** Comparative analysis of HeV and NiV VNT results from bats sampled in Australia, PNG, Indonesia and East Timor.

Location	Species	Total sampled	Total positives	*%* *positive*	Hendra Virus reactivity	*%* *positive*	Nipah Virus reactivity	*%* *positive*	Equivocal titre	*% positive*
Australia	P. conspicillatus	180	119	*66.1%*	52	*43.7%*	8	*6.7%*	59	*49.7%*
PNG	All species	77	46	*59.7%*	4	*8.7%*	3	*6.5%*	39	*84.8%*
PNG	P. alecto	15	5	*33.3%*	1	*20.0%*	0	*0.0%*	4	*80.0%*
PNG	P. conspicillatus	53	34	*64.2%*	2	*5.9%*	2	*5.9%*	30	*88.2%*
PNG	P. neohibernicus	9	7	*77.8%*	1	*14.3%*	1	*14.3%*	5	*71.4%*
PNG	D. magna	8	1	*12.5%*	0	*0%*	1	*12.5%*	0	*0*
Indonesia	All species	60	17	*28.3%*	7	*41.2%*	0	*0.0%*	10	*58.8%*
Indonesia	P. alecto	45	15	*33.3%*	6	*40.0%*	0	*0.0%*	9	*60.0%*
Indonesia	A. celebensis	15	2	*13.3%*	1	*50.0%*	0	*0.0%*	1	*50.0%*
East Timor	P. vampyrus	51	22	*43.1%*	1	*4.5%*	19	*86.4%*	2	*9.1%*

Note: Only those species for which as least one seropositive VNT result was obtained and for which VNT testing against both HeV and NiV was conducted are included in this table. Hence negative results are not shown for *P. macrotis* (n = 3), *R. amplexicaudatus* (n = 30) and *M. minimus* (n = 21).

### Papua New Guinea

Fruit bats were sampled in Western Province (n = 56) and Madang Province (n = 53). None of the animals tested positive on RT-qPCR for HeV or NiV RNA from samples of blood, urine or saliva.

Three *Pteropus* species showed seroreactivity to henipaviruses on VNT. These were: *P. alecto* 5 of 15 (33%, 95% CI 9–57), *P. neohibernicus* 7 of 9 (78%, 95% CI 51–100) and *P. conspicillatus* 34 of 53 (64%, 95% CI 51–77) (see Table 2). Of the 46 *Pteropus* bats returning positive results on VNT, 4 (8.7%) suggested exposure to HeV, 3 (6.5%) exposure to NiV, and 39 (84.8%) showed equivocal titres (Figure 1).

Eight *Dobsonia magna* were sampled; one showed a positive VNT to NiV (titre 1∶10) but was negative for HeV antibodies. Twenty-one *Macroglossus minimus* and three *Pteropus macrotis* were sampled and none showed seroreactivity to either HeV or NiV on VNT.

### East Timor

Fruit bats were sampled in Cova Lima Province (n = 82). Species sampled were *P. vampyrus* (n = 51), *Pteropus griseus* (n = 1), *Rousettus amplexicaudatus* (n = 30) and *Dobsonia peronii* (n = 1).

NiV RNA was detected by RT-qPCR in the blood of one *P. vampyrus* with a CT value of 43 and in the saliva of four *R. amplexicaudatus* (CT values 38.5, 41, 39 and 39). No HeV RNA was detected in any of the samples tested. NiV RNA was also detected in the urine of one *R. amplexicaudatus* by nested RT-PCR and a 357 nucleotide fragment sequence was obtained from the Nucleocapsid-gene. This sequence showed 100% homology to Malaysian NiV isolates from a *Pteropus hypomelanus* (Genbank accession number AF376747), pig (Genbank accession number AJ627196) and human (Genbank accession number NC_002728); 98% homology to a NiV isolated from *Pteropus lylei* in Cambodia (Genbank accession number AY858110); and 93% homology to a NiV isolate from a human in Bangladesh (Genbank accession number AY988601).

Twenty-two of 51 (43%, 95% CI 30–57) *P. vampyrus* sampled showed positive results on VNT: 1 (4.5%) indicated exposure to HeV, 19 (86.4%) indicated exposure to NiV, and 2 (9.1%) showed equivocal titres (see Table 2; Figure 1). None of the 30 *R. amplexicaudatus* sampled showed positive results on VNT, although seroreactivity to NiV was detected in 6 of 23 animals on Luminex serology (results not shown).

### Indonesia

Fruit bats were sampled in Sulawesi (n = 59) and Sumba (n = 3). Species sampled were *Acerodon celebensis* (n = 15), *Pteropus alecto* (n = 45), *Rousettus amplexicaudatus* (n = 1) and *D. peronii* (n = 1).

Henipavirus RNA was detected by generic RT-qPCR in eight pooled urine samples from *P. alecto* and *A. celebensis* from Sulawesi, with CT values of 31–34. Henipavirus RNA was detected in the urine sample from one *P. alecto* from Sumba by RT-qPCR with a CT value of 28. These samples testing positive for henipavirus RNA by RT-qPCR; all tested negative for both HeV and NiV RNA by type-specific RT-qPCR.

Fifteen of the 45 (33%, 95% CI 20–47) *P. alecto* and 2 of the 15 (13%, 95% CI 0–31) *A. celebensis* sampled showed positive results on VNT: 7 (41.2%) indicated exposure to HeV, 0 indicated exposure to NiV, and 10 (58.8%) showed equivocal titres (Table 2; Figure 1).

Attempts at viral isolation from samples yielding positive PCR results were all unsuccessful.

## Discussion

The aim of this study was to determine the occurrence and diversity of henipaviruses in fruit bat populations in the regions of northeast Australia, New Guinea (Papua New Guinea) and Wallacea (Indonesia and East Timor). Nipah virus has had a much greater impact than HeV on human and domestic animal health to date and hence we proposed to determine if NiV occurs east of the Wallace Line. We also aimed to determine if henipaviruses circulated in fruit bat species other than those of the Genus *Pteropus* in the Australasian region. We used serological and molecular approaches to determine the presence of henipaviruses in fruit bat populations and attempted to identify the species of virus when evidence of henipaviruses was detected.

### Australia

At the time of this study was initiated, the detection of henipavirus RNA in wild bats was a rare event, thus the sampling of a population near Townsville just one month after a nearby spillover event [13], was an opportune time to enhance the likelihood of detecting virus in a bat population. Results from the sampling of 64 *P. alecto* at a colony just 1 km from where a horse had contracted HeV near Townsville one month previously confirmed the presence of HeV in this bat population with the positive detection of HeV RNA in blood, urine and saliva from a single individual. This finding was supported by the detection of antibodies to HeV by VNT at a seroprevalence of 44% although comparative testing for NiV antibodies was not performed.

The sampling of 180 *P. conspicillatus* near Cairns (where three spillover events of HeV to horses had occurred in the past [4], failed to yield any PCR positive samples to henipavirus RNA. Nevertheless, 67% of the bats had antibodies to henipaviruses, with 43.7% of these indicating exposure to HeV, 6.7% indicating exposure to NiV and 49.6% showing equivocal titres (see Figure 1). Given the previous cases of HeV in horses in this area over a period of eight years and the subsequent frequent detection of HeV from fruit bats in this area by Field et al. [36], it appears highly likely that HeV is endemic in this fruit bat population. However the high proportion with equivocal titres (49.6%) and the small proportion of samples returning comparative titres indicating NiV exposure (6.7%) are perplexing. Possible explanations for the equivocal titres and those that suggest exposure to NiV include: that the immunological response may vary among individual bats such that a fourfold or higher titre to HeV was not present in all bats following exposure to HeV; exposure of the sampled bats to a different henipavirus from that of HeV; co-infection, or subsequent infection, of bats with HeV and another henipavirus had taken place; the HeV strain used in the VNT is antigenically different to the HeV strain that the bats had been exposed to. The recent detection of Cedar virus in Australian bats may support the second and third explanations above [37].

### Papua New Guinea (PNG)

Henipavirus RNA was not detected in samples from any of the bats from PNG despite expending considerable effort to maximise the likelihood of detection of viral RNA. This included: the collection of samples into a commercial RNA preservative (“RNA-Later”, Qiagen, Doncaster, Australia), as well as viral transport medium to improve preservation of RNA, the use of a dry-shipper to hold samples at −150°C immediately following collection until processing at the laboratory, and the screening of samples with a generic “henipavirus” RT-qPCR prior to HeV and NiV specific RT-qPCR testing.

The comparative serology of bats from PNG showed a very high proportion (84.8%) with equivocal titres to HeV and NiV on VNT, with similar and small proportions indicating exposure to HeV (8.7%) and NiV (6.5%) (see Figure 1). Further data are required to determine which species of henipavirus occur in these populations, but it is clear that henipaviruses are indeed present, though their threat to animal and public health remains unclear.

None of 21 *Macroglossus minimus* sampled showed seroreactivity to either HeV or NiV. This may provide 95% statistical confidence that the *M. minimus* population does not support henipavirus infection, assuming a minimum seroprevalence in the population of 14% if virus were present and representative sampling of the population. This may suggest that henipaviruses do not circulate in this species, or at least not at the seroprevalence usually detected in *Pteropus* species. Although *M. minimus* has a very large distribution from Australia and New Guinea, through the Indo-Malayan archipelago to mainland Asia, these results suggest that it is unlikely to act as a reservoir host if virus infects these animals. None of three sampled *Pteropus macrotis* individuals was seropositive to henipaviruses, but the small sample size limits meaningful interpretation of these findings.

### East Timor

Several detections of NiV were made by PCR in the samples from East Timor. These positive results were obtained both by RT-qPCR and by nested RT-PCR. The detection of NiV RNA by nested PCR from a urine sample from *R. amplexicaudatus* allowed amplification and sequencing of a 357 base pair RNA fragment and comparison to published NiV nucleotide sequences. The sequence showed 100% homology with the nucleotide sequence of NiV isolates from Malaysia. This finding is thus consistent with the distribution of *R. amplexicaudatus* and *P. vampyrus* in both Timor and Malaysia. The detection of NiV RNA by RT-qPCR in saliva samples from three other R. *amplexicaudatus*, albeit at high CT values (38.5, 41, 39 and 39), adds weight to the contention that NiV was circulating in this population of bats at the time of sampling. The detection of NiV in the blood of one of the *P. vampyrus* sampled with a CT value of 43 suggests an extremely small amount of viral RNA may have been present and is of dubious significance when considered in isolation. However when the comparative serology results are considered from the same population of animals indicating exposure to NiV in 86.4% (see Table 2 and Figure 1) of animals, the body of evidence supporting the presence of NiV in fruit bats in East Timor is strong.

The presence of NiV in *P. vampyrus* on Timor is not completely unexpected given that NiV has been isolated from this species of bat on mainland Asia [7], and that this species also occurs on Sumatra, Java and the Lesser Sunda Islands, including Timor [38]. A genetic study of *P. vampyrus* indicates a high level of gene flow among populations throughout their range [39], and satellite telemetry has shown *P. vampyrus* to fly between peninsular Malaysia and Sumatra [40], indicating the potential of viral transfer from one population to another of this species. The finding of NiV RNA in *R. amplexicaudatus* is surprising as henipaviruses have rarely been found in bat genera other than *Pteropus*, although antibodies to henipaviruses have been found in a related species *Rousettus leschenaulti*, in China and Vietnam [41], [42]. In the study by Li et al. [41], although five of 16 individuals sampled from one location gave a positive response on ELISA and western blotting assays, these sera did not neutralise HeV or NiV on VNT. This is consistent with our findings in *R. amplexicaudatus* of a lack of neutralising seroreactivity on VNT, but some seroreactivity on Luminex serology (data not shown). This may be due to a different immune response to henipavirus infections in non-Pteropid bats where a low level of neutralising antibodies are produced that are difficult to detect in current assay systems [41].

### Indonesia - Sulawesi

Eight pooled urine samples containing urine from both *P. alecto* and *A. celebensis* showed positive results on a RT-qPCR with primers designed for a region of the nucleocapsid gene that is conserved among published HeV and NiV sequences. The CT values of the positive samples were all within the range of 31–34 cycles. These samples were then tested with a HeV specific and a NiV specific RT-qPCR with negative results. This suggests the virus present in these urine samples was a henipavirus other than HeV or NiV. The comparative serology from these animals showed the highest proportion returning equivocal titres (58.8%) and the rest indicating exposure to HeV (41.2%) and none indicating exposure to NiV. Possible explanations of these findings include infection of the *P. alecto* and *A. celebensis* populations of north Sulawesi with a Hendra-like virus (that differs in nucleotide sequence at the primer binding site of the HeV RT-qPCR), or previous exposure to HeV and current infection with a Hendra-like virus.

### Indonesia - Sumba

One *P. alecto*, an adult male, was captured and sampled on Sumba. Henipavirus RNA was detected in its urine using the RT-qPCR with primers designed for a conserved region of the nucleocapsid gene at a CT of 28. Subsequent testing of the urine sample with HeV and NiV specific RT-qPCR was negative for both viruses. This animal showed equivocal titres to HeV and NiV on VNT. These findings are consistent with infection of this animal by a henipavirus that differs in nucleotide sequence from HeV and NiV but is closely related to both viruses in terms of nucleotide sequence and in elicitation of antibodies that neutralise HeV and NiV at similar titres.

### Conclusions

This study showed clear evidence for the presence of NiV east of Wallace’s Line in East Timor, although it was not detected in individuals sampled from Sulawesi, Sumba or New Guinea (see Figure 1). This extends the range of areas from which NiV has been detected by PCR from peninsular Malaysia by over 2,500 km to the southwest to the island of Timor. However the results from Sulawesi and Sumba suggest NiV may not be present throughout the intervening area. Rather, the distribution of NiV may be linked to the presence of specific fruit bat species, particularly *P. vampyrus*.

We also found clear evidence of the presence of henipaviruses in non-Pteropus species in Australasia: *Acerodon celebensis* in Sulawesi and *Rousettus amplexicaudatus* in East Timor. A single seropositive result in *Dobsonia magna* from Papua New Guinea adds to several other detections of henipavirus antibodies in bats of this genus [20].

A major finding in this study was evidence for non-NiV, non-HeV henipaviruses in the region. We found molecular evidence for such viruses in Sulawesi and Sumba, with samples positive in a generic henipavirus PCR assay but not in NiV or HeV specific assays. In addition, we found serological indication for such viruses in those two locations, and also in Australia, PNG, and to a lesser extent in East Timor, with samples showing equivocal neutralising antibody titres against both NiV and HeV. While HeV and NiV are the only recognised pathogenic henipavirus species, there is accumulating evidence that other henipaviruses exist [37], [43].

As with other emerging infectious diseases of wildlife, serological and virological diagnostic capabilities are limited due to incomplete understanding of the diversity and relatedness of these pathogens (e.g. level of cross reactivity). Further studies utilising enhanced genome detection methods in areas where equivocal serological results are obtained are required to elucidate the risk posed by henipaviruses.

This study, in combination with the serological evidence of henipavirus infection in *P. vampyrus* from Sumatra, Java and Borneo [17], [18], has shown that henipaviruses occur in fruit bats widely across the Sunda Shelf, Wallacea and New Guinea. Future work could be fruitfully directed towards further characterisation of the diversity of henipaviruses in Wallacea and New Guinea where novel henipaviruses may occur. The evidence presented here suggests such viruses do exist, though the threat they may pose to human and animal health remains unclear. Also further investigation of the role of non-Pteropus fruit bats in the ecology of henipaviruses is indicated, particularly members of the genera *Rousettus* and *Acerodon*.
